# A265 BARRIERS TO DIETARY MODIFICATION IN INFLAMMATORY BOWEL DISEASE (IBD): A MIXED-METHODS ASSESSMENT OF PATIENT PERCEPTIONS

**DOI:** 10.1093/jcag/gwad061.265

**Published:** 2024-02-14

**Authors:** J Szeto, C V Noejovich, R Verma, P Miranda, M Pinto-Sanchez, E Verdu, D Armstrong

**Affiliations:** Medicine, McMaster University Faculty of Health Sciences, Hamilton, ON, Canada; Medicine, McMaster University Faculty of Health Sciences, Hamilton, ON, Canada; Medicine, McMaster University Faculty of Health Sciences, Hamilton, ON, Canada; Medicine, McMaster University Faculty of Health Sciences, Hamilton, ON, Canada; Medicine, McMaster University Faculty of Health Sciences, Hamilton, ON, Canada; Medicine, McMaster University Faculty of Health Sciences, Hamilton, ON, Canada; Medicine, McMaster University Faculty of Health Sciences, Hamilton, ON, Canada

## Abstract

**Background:**

Many patients living with IBD identify diet as a key factor in managing their disease, symptoms and general health, and many report implementing dietary restrictions in response to disease activity and symptoms. Despite increasing data on the role of diet, IBD patients face a variety of challenges that can compromise adherence to dietary recommendations in clinical practice.

**Aims:**

To identify IBD patients’ perceptions regarding barriers to dietary modification and to understand their experiences and expectations of dietary advice from gastroenterologists (GI) or dietitians (RD).

**Methods:**

A mixed-method qualitative data collection strategy with semi-structured focus group and individual one-on-one interviews moderated by a clinical psychologist over a web-based, video communication platform (Zoom). Adult IBD patients (between 18 to 75 years old) attending the McMaster University Medical Centre IBD Clinic were invited to join a focus group consisting of 2-6 individuals or a one-on-one interview. All participants were asked to complete a demographics survey (REDCap) before the session. Recorded audio files for all sessions were transcribed, de-identified and reviewed for accuracy by 2 reviewers with an independent adjudicator to resolve discrepancies followed by thematic analysis (NVIVO).

**Results:**

Between May to December 2022 and May 2023, 38 of 90 invitees took part in 11 focus groups and 9 chose individual interviews. Most participants (mean age 42 years; 60% female) were Caucasian (87%); 42% had a self-reported history of mental health disorders. Mean IBD duration was 16 years (min-max: 0.5–44 years); 73% were in remission and 68% had Crohn’s disease. Thematic analysis identified 5 primary and 11 secondary barriers to dietary adoption (Table).

Participants reported positive and negative experiences with dietary advice from GIs and RDs; expectations included GI referral to a specialist RD and integration of an RD into the health care team.

**Conclusions:**

IBD patients report multiple, varied barriers to dietary adoption and identify a need for improved access to dietary advice and other resources, including integration of RDs into primary and IBD Clinic care teams. The identification of multiple, varied patient-reported barriers offers an opportunity to develop personalized dietary advice for IBD patients to enhance health, well-being and quality of life.

Thematic map of barriers experienced by patients when adopting dietary modifications

**Funding Agencies:**

Farncombe Family Digestive Health Research Institute; Douglas Family

**Table:** 

Primary Barriers	Secondary Barriers
Difficulty identifying foods or diets affecting IBD	Lack of guidance in identifying foods or diets Difficulty correlating symptoms with diet
Barriers to accessing IBD diet foods	Limited availability of IBD-friendly food options Expense of speciality food items
Psychological Factors	Anxiety in relation to food Aversive attitude to food
Personal struggles with diet adherence	Challenges with diet protocol complexity Changing habits and lifestyle Avoiding food cravings
Social and family challenges in dietary implementation	Stress and pressure when eating in social settings Challenge integrating family’s needs into diet

